# Application of the ASCO value framework to evaluate the clinical and economic value of enzalutamide and apalutamide in the early stages of prostate cancer in Colombia

**DOI:** 10.3332/ecancer.2023.1614

**Published:** 2023-10-23

**Authors:** Martín Romero, Andrea Díaz, Karen Sánchez, Sandra Amaya, Fabián Godoy, David Rodríguez

**Affiliations:** 1 Grupo Proyectame S.A.S. Bogotá, D.C. 111121, Colombia; 2 Astellas Farma Bogotá, D.C. 111121, Colombia; 3 Instituto Nacional de Cancerología E.S.E Bogotá, D.C. 111121, Colombia; a https://orcid.org/0000-0001-6158-6090; b https://orcid.org/0000-0002-4007-1976; c https://orcid.org/0000-0002-1469-9655; d https://orcid.org/0000-0003-3524-2889; e https://orcid.org/0000-0002-4814-5255

**Keywords:** prostatic neoplasms, prostatic neoplasms castration-resistant, androgen antagonists, technology assessment biomedical, drug costs, costs and cost analysis

## Abstract

**Introduction:**

Prostate cancer has increased in recent years, increasing the costs associated with its treatment. Second-generation oral antiandrogens have emerged as an attractive therapeutic option.

**Objective:**

To compare the health value provided by enzalutamide and apalutamide, by evaluating two stages of prostate cancer: non-metastatic castration-resistant prostate cancer (nmCRPC) and metastatic hormone-sensitive prostate cancer (mHSPC).

**Methods:**

To establish, through the American Society of Clinical Oncology (ASCO) value framework, a contrast between two technologies in two stages of prostate cancer. The monthly cost of the two technologies was calculated according to the current price regulation norm in Colombia.

**Results:**

Enzalutamide showed a higher net health benefit score compared to apalutamide for both nmCRPC (48.33 versus 33.46) and mHSPC (52.0 versus 40.75). The cost per net health benefit point for the nmCRPC stage was $214,723 Colombian Pesos (COP) ($54.84 USD) with enzalutamide compared to $291,925 COP ($74.56 USD) with apalutamide, and for the mHSPC stage was $199,692 COP ($51.00 USD) with enzalutamide and $239,701 COP ($61.22 USD) with apalutamide.

**Conclusion:**

After comparing enzalutamide versus apalutamide in the nmCRPC and mHSPC stages through the ASCO value framework, enzalutamide showed a more prominent net clinical benefit and a lower investment per point awarded.

## Introduction

Prostate cancer accounts for 0.86% of total deaths in the world [1]. It is the second most frequent cancer in men worldwide and the most frequent in Colombia [2]. Although the incidence rates vary by country, there has been an overall increase since 1990 due to the screening programmes. However, mortality rates have fallen due to a combination of early disease detection and the use of curative-intent treatments [3].

According to the official data of the High-Cost Diseases Office in Colombia, there was an estimated prevalence of 164 out of every 100,000 men, and a mortality rate of 9.03/100,000 in 2020, thus making this the cancer with the highest mortality in men [4]. Prostate cancer is relatively slow-growing, which makes it possible to determine the different stages based on its response to androgen deprivation therapy (ADT) (hormone-sensitive/castration resistant) and the presence or absence of metastasis (metastatic/non-metastatic). Since 1941, it has been recognised that prostate cancer progression is driven by androgen levels. The mainstay of treatment is to therefore maintain castrate levels of testosterone (<50 ng/dL) either surgically or chemically [5]. It is estimated that up to one-third of patients will progress to advanced stages [6].

In the last 5 years, there has been significant progress in the treatment of prostate cancer with the emergence of second-generation anti-androgens as a reinforcement to the classic ADT [7]: enzalutamide, apalutamide and darolutamide. These drugs inhibit the binding of androgens to the androgen receptors, the nuclear translocation of activated androgen receptors and impairs activated androgen receptor-DNA binding [8]. They have demonstrated increased clinical benefit in survival when added to the treatment [9–11].

The notion of value-based healthcare goes back to Porter and Teisberg [12], who defined it as the health outcomes achieved per dollar spent. Outcomes refer to multidimensional concepts that jointly determine the success in meeting patient needs [12]. Value-based healthcare is measured and managed according to the fulfilment of defined needs to clearly define the outcomes against the costs [13]. The primary objective is to improve the outcome-cost ratio through the provision of interventions aimed at specific patient segments [14]. Value-based healthcare analyses have increasingly been recognised as a decision support tool. These analyses propose a more comprehensive approach, which seek to include elements beyond cost-effectiveness [15].

The following formulation provides a general overview of the value concept described by Porter [16]. However, it is more of a conceptual formulation than a mathematical equation [14, 16].


$$\mathrm{Value}=\frac{\mathrm{Health}\;\mathrm{results}}{\mathrm{Costs}\;\mathrm{in}\mathrm{curred}\;\mathrm{to}\;\mathrm{deliver}\;\mathrm{the}\;\mathrm{results}}.$$

Defining the value concept and the elements that should be considered in its analysis presents a challenge clinically, pharmacologically and in health economics. Various organisations have proposed value-based healthcare assessment models, using two or more tools known as value assessment frameworks (VAF) [17]. Various VAF tools are used in cancer treatment, like those proposed by the *American Society of Clinical Oncology* (ASCO) [18]; the *National Comprehensive Cancer Network* (NCCN) and the *European Society of Medical Oncology* (ESMO) [19], among others. These generally come from expert assessment processes, which determine the attributes that should be considered and their relevance [20].

The ASCO value framework was designed to help doctors and patients assess the value of a cancer pharmacological treatment, compared to a standard treatment [21], to facilitate joint decision-making between oncologists and patients [22]. This generates a net health benefit score for treatments using data obtained from randomised controlled trials. The value framework outlined by this agency includes clinical efficacy, safety profile, long-term survival, palliation, quality of life and treatment-free interval [22]. This article aims to analyse the value of enzalutamide and apalutamide in prostate cancer treatment, especially in the non-metastatic castration-resistant prostate cancer (nmCRPC) and metastatic hormone-sensitive prostate cancer (mHSPC) stages.

## Methodology

### ASCO framework

Various elements are considered in calculating the net health benefit of the assessed techniques, which is a score calculated using measures of clinical benefits, toxicity and bonus points.

#### Clinical benefit

The calculation is based on the hazard ratio (HR) for deaths. When this is not available, other estimates must be used, including the median overall survival (OS), the HR for progression-free survival (PFS), the median PFS or response rate (RR) [18].

#### Toxicity

The calculation is based on the frequency of Grade 1 to 4 adverse events, as defined in the Common Terminology Criteria for Adverse Events. For each Grade 1 or 2 adverse events, a score of 0.5 is assigned if the frequency is <10% and 1 if it is ≥10%. For Grade 3 or 4, a score of 1.5 is assigned if its frequency is <5% or 2.0 if it is ≥5%. The points for each technique and the percentage difference between each therapy are then calculated. The percentage difference is multiplied by 20 to obtain an overall toxicity score. If the test regimen is more toxic, there will be a negative score [18].

#### Bonus points

The sum of four elements is calculated, each of which is assigned an individual score: 1) tail of the curve: up to 20 points are assigned if an increase greater than 50% is observed in the survival for the assessed treatment compared to the standard treatment on the curve point on the *x*-axis representing double the median OS of standard treatment; 2) palliation of symptoms receives 10 points if a statistically significant reduction in cancer-related symptoms is reported; 3) quality of life receives 10 points if a significant improvement is reported in the quality of life of patients undergoing treatment; and 4) treatment-free interval receives 10 points if a statistically significant improvement is shown in the treatment-free interval [18, 23].

In the ASCO framework, advanced disease costs are given per month of treatment and consider the cost of drug procurement [21]. In the Colombian health system, this is fully covered by insurance companies.

### Literature review

To obtain information in accordance with the ASCO framework directives, a systematic literature review was conducted on MEDLINE in March 2022. For the value analysis, trials that specifically included apalutamide (ERLEADA®) and enzalutamide (XTANDI®) were selected. These trials were used as primary sources to establish the relevant value frameworks (Appendix 1: Search strategies, see https://doi.org/10.6084/m9.figshare.24221227.v1).

### Costs

The cost data for each of these drugs was calculated based on the current Circular 13/2022 drug pricing regulation legislation [24], which sets the maximum regulatory drug prices in Colombia. The dosage was calculated in accordance with clinical trials and clinical practice guidelines [25–27].

## Results

**Figure 1.**
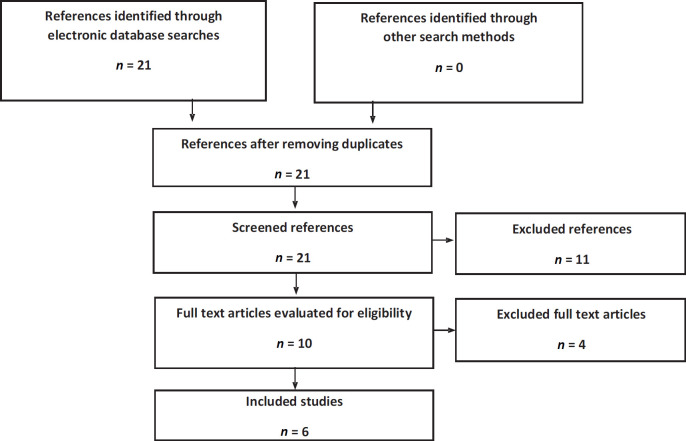
Flowchart of search, screening and selection of evidence in nmCRPC. Source: Prepared by the authors.

For the literature review in the nmCRPC setting, six articles were obtained from publications on two clinical trials; PROSPER and SPARTAN (Figure 1). The PROSPER trial assessed the impact of ADT + enzalutamide compared to ADT + placebo. Three publications were obtained from this clinical trial: an interim analysis [28], a final mortality analysis [29] and a quality of life study [26]. The SPARTAN assessed the impact of apalutamide + ADT compared to ADT + placebo. Three publications were obtained from this clinical trial: an interim analysis [30], a final survival analysis [31] and a quality of life study [27].

**Figure 2.**
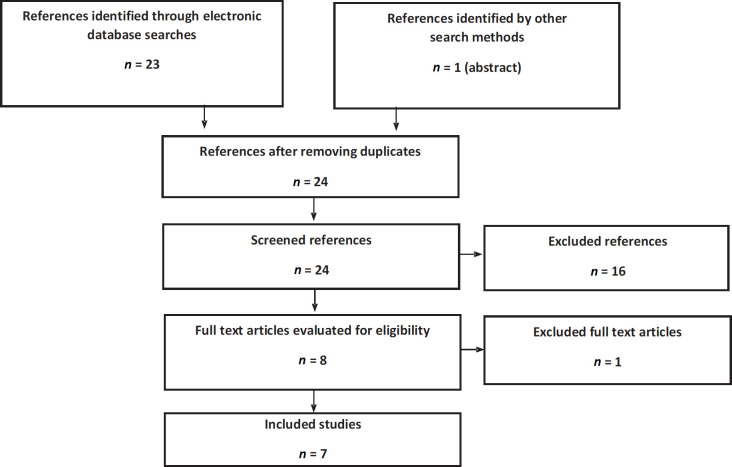
Flowchart of search, screening and selection of evidence in mHSPC. Source: Prepared by the authors.

For the literature in the mHSPC setting, seven articles were obtained from two clinical trials: ARCHES and TITAN (Figure 2). The ARCHES trial assessed the impact of enzalutamide + ADT compared to ADT + placebo. There were three peer-reviewed publications: an interim analysis [32], an abstract with final mortality results [33] and a quality of life study [34]. The TITAN trial assessed the impact of apalutamide + ADT compared to ADT + placebo. Four publications were obtained: an interim analysis [35], a final survival analysis [36], a quality of life study [37] and an additional pain and fatigue analysis [38]. The characteristics of these trials are outlined in Appendix 2.

Costs for the assessed treatments come from Circular 13/2022 [24], which sets the maximum regulatory drug prices. The costs set out below were monthly estimates and apply to the two stages of interest. The dosage was calculated in accordance with clinical practice guidelines [25] (Table 1).

### Implementation of ASCO VAF

#### nmCRPC

**Enzalutamide: PROSPER *t*rial.** The use of enzalutamide improves OS compared to placebo (HR 0.73; CI 95%, 0.61–0.89), thus generating a clinical benefit score of 27 points [29]. Enzalutamide received a toxicity score of −4.64, thus making it a more toxic treatment than placebo.

The percentage of metastasis-free patients was 50% greater compared to the control at 29.4 months, thus resulting in 16 tail of the curve bonus points [29]. When assessing the quality of life, no statistically significant difference was found in the overall FACT-P and EQ-5D-5L scores. However, according to FACT-P, significant changes were observed for enzalutamide in social and family well-being with a mean least square difference of 0.94 (CI 95%, 0.02–1.85) (*p* = 0.045) [26], thus resulting in 10 additional points. Additionally, a slower deterioration in the quality of life associated with the disease is reported due to the use of enzalutamide. No bonus points were assigned for the palliation of symptoms because no improvements were evident. Treatment-free interval data were not available. The net health benefit resulting from the sum of all the elements was 48.36 out of 180 possible points.

**Apalutamide: SPARTAN *s*tudy.** Analysis of apalutamide plus TPA demonstrated a baseline OS of HR 0.78 (CI 95%, 0.23–0.35) yielding a clinical benefit score of 22. −4.54 toxicity points were calculated as apalutamide was the therapeutic alternative that reported the most adverse effects [31].

16 bonus points were assigned to the tail curve because the proportion of subjects free of metastasis was identified to be 50% higher than the control at 32.4 months. SPARTAN reported that the quality of life remained similar for the two groups throughout the study, FACT-P and EQ-5D-3L specific scales did not show significant differences in overall scores or domains [27]. The study does not offer information about the palliation of symptoms of treatment-free interval, for which these bonus points are not awarded. The net health benefit was 33.46 out of 180 possible points.

It was estimated that in the nmCRPC stage, in order to acquire a health benefit point, $214,723 Colombian Pesos (COP) ($54.84 USD) must be invested in enzalutamide; while $291,925 COP ($74.56) must be invested in apalutamide (Table 2).

#### mHSPC stage

**Enzalutamide: ARCHES *s*tudy.** An OS of HR 0.66 (CI 95%, 0.53–0.81) was reported for enzalutamide [32, 33], obtaining a total of 34 points of clinical benefit. Toxicity calculations resulted in a score of −2.0 because enzalutamide has a higher frequency of adverse effects [32].

No tail curve points were assigned because the point on the curve that was twice the placebo median was not reached. For the quality of life outcome, no statistically significant changes were reported for the overall scores of the QLQ-PR25, FACT-P, BPI-SF, EQ-5D-5L scales and their domains. However, enzalutamide was reported to significantly delay the time of first clinically significant deterioration for the onset of worse pain, for the severity of pain and for visual analogue scale score EQ-5D-5L [34]. With regard to the points for palliation of symptoms, a delay in the first symptomatic musculoskeletal effect was reported [33], on adding the two attributes, 20 bonus points were assigned and no points were assigned for the treatment-free interval as they were not available. A net health benefit of 52 out of 180 possible points was obtained.

**Apalutamide: TITAN *s*tudy.** In the case of apalutamide plus TPA when compared with placebo plus TPA, an OS of HR 0.67 (CI 95%, 0.51–0.89) [36] is seen, generating a clinical benefit of 33 points. The toxicity calculations resulted in a score of −2.25 as apalutamide was more toxic than the placebo [35].

No tail curve points were assigned because the point on the curve that was twice the placebo median was not reached. There were no statistically significant changes for overall scores of the FACT-P, BPI-SF, EQ-5D-5L scales and their domains, no significant delay in the worsening of the FACT-P and EQ-5D-5L scores was reported either, and maintenance of the quality of life score is concluded during the study for which no quality of life points are assigned [37]. With regard to palliation, a complementary study on pain and fatigue found a statistically significant prolongation in the median time to event for pain deterioration and other pain-related outcomes of interest [38], for which 10 palliation points were awarded. The treatment-free interval data was not available, yielding a net health benefit of 40.75 out of 180 possible points.

It is estimated that in the mHSPC stage, $199,692 COP ($51.00 USD) must be invested in order to obtain a health benefit point on using enzalutamide, while $239,701 COP ($61.22 USD) must be invested for each point, on using apalutamide (Table 3).

Enzalutamide obtained a higher point in the net health benefit than apalutamide, and for both nmCRPC (48.33 versus 33.46) and nmHSPC (52.0 versus 40.75). The cost per point of the net health benefit for the nmCRPC stage was 214,723 COP ($54.84 USD) for enzalutamide versus 291,925 COP ($74.56 USD) for apalutamide; while for the mHSPC stage it was 199,692 COP ($51.00 USD) for enzalutamide and 239,701,348 COP ($61.22 USD) for apalutamide. In both stages, enzalutamide requires a smaller investment to acquire one point in net clinical benefit.

## Discussion

Due to the rapid emergence of new cancer therapies and their high costs, new models have been formulated to establish the appropriate price for these; it has been proposed that the therapeutic alternatives that represent the greatest clinical benefits must be those that are assigned the highest prices and profit margins, with the aim of encouraging research and innovation. Thus, the VAFs emerge as strategies to evaluate the overall benefits offered by each treatment [39].

Each existing VAF offers its own approach and methodology, such that there are no two frameworks which use the same subdomains, formulas or scoring scales [22, 40]. The decision to use the ASCO framework for this study was based on an analysis of the different frameworks available for cancer. The NCCN framework is a more subjective tool for doctor-patient decision making since it allows the inclusion of data such as expert opinion, clinical experience, case reports or unpublished information [40], while the ESMO and ASCO frameworks examine similar features, however, ESMO was discarded for not including costs. One could even think that there is a need for a specific framework for prostate cancer that incorporates specific characteristics of the neoplasm, as it could have an effect on sexual and urinary function.

Wong *et al* [41], did a value analysis of enzalutamide using the frameworks proposed by ASCO and ESMO, noting that with the ASCO methodology, enzalutamide represents a net health benefit of 62 or 64 points for the mCRPC stage and 17 or 59 points for the mHSPC stage, and they, therefore, concluded that the clinical benefit was less in the early stages of the disease [41]. The variation of points was based on the clinical study used, in the case of the mHSPC, they obtained a score of 17 on using the ARCHES study and 59 using the ENZAMET study [42]. The difference with the findings reported in this analysis (52 points) lies in the fact that Wong *et al* [41] took the survival HR reported in the interim analysis dated 2019 [32] (HR 0.81; CI 95%, 0.53–1.25; *p* = 0.3361), although both sets of data came from the ARCHES study, they were measured at different times, as the data used by Wong *et al* [41] was still premature. Additionally, the authors did not award any bonus points for palliation of symptoms or quality of life, although there was no significant difference in the overall QLQ-PR25, FACT-P y EQ5D-5L scores if a delay in the appearance of pain and in the first clinically significant deterioration measured with EQ5D-visual analogue scale (VAS) was reported. This shows that the framework has an important subjective component since it depends on the interpretation of the qualifier, especially for the bonus points. In spite of the differences found, it can be affirmed that in both Wong *et al*’s [41] study and this analysis, enzalutamide represents a substantial clinical benefit for those evaluated, since it exceeds the 45 cut-off points established [43].

The study by Shah-Manek *et al* [40] that applied the ASCO framework for enzalutamide in the metastatic castration-resistant stage (mCRPC), found that the net health benefit calculated was between 45 and 71 points and that the cost per point of net health benefit was between 120 and 190 USD, depending on the clinical trial used. The findings are aligned with what Wong *et al* [41] reported for this study. On the other hand, this same analysis exercise has been performed through other value frameworks, as in the case of NCCN (Appendix 4). Although the two frameworks differ widely in their methodology and attributes evaluated, the result was in favour of enzalutamide in both cases.

In our study, the advantage of enzalutamide is mainly mediated by the bonus points, especially the improvement in the outcomes of quality of life and palliation of symptoms. Additionally, from the value shown in this study, enzalutamide has practical benefits, such as the fact that in the castration-resistant scenario, it is approved for metastatic and non-metastatic stages, therefore, regardless of administrative delays in proving the presence or absence of metastasis, the medication will be effective and will not need to be changed.

Within the limitations of this study, we can highlight that the data come from clinical trial-type studies, that is to say, it is carried out in controlled environments that could differ from behaviour in real life. Additionally, due to the specific characteristics of the framework value used, the total costs of treatment are not taken into account. Although in Colombia, the patient does not incur additional expenses for the acquisition of technology given that the health system covers this, it would be interesting to analyse other out-of-pocket expenses which could be derived from each treatment. It is important to highlight that the data supporting the value framework did not come from head-to-head studies between enzalutamide and apalutamide as a desirable series, rather the studies done evaluate the molecules against TPA + placebo which causes heterogeneity in terms of what is reported and comparability is compromised. Finally, an important subjective component was confirmed in the evaluation of some of the attributes.

This study represents a contribution to the analysis of therapeutic alternatives in prostate cancer in the early stages, in order to understand the benefits of oral antiandrogens from a perspective that supports negotiation and payment models between actors, since, to date, no analysis of this type has been found in the literature for Colombia. Similarly, there is evidence of the opportunity to carry out this type of analysis for other stages of cancer, such as advanced stages for which there are also high-quality trials of these two molecules. It is worth mentioning that the outlook of current treatment could change in the short/medium term, with the possible entry of trimodal therapies [44, 45] for the hormone-sensitive stage.

## Conclusion

Enzalutamide and apalutamide are the latest generation of oral androgen receptor inhibitors, i.e., they do not require additional resources for their administration. In general, both technologies have performed similarly in terms of effectiveness, with either technology improving the effectiveness of TPA alone. However, when analysed from a wider perspective such as value frameworks, enzalutamide was found to represent higher net economic benefits than apalutamide for the two stages analysed in Colombia.

## Appendices and supplementary information

The appendices and supplementary information are on FigShare at the following link:

https://doi.org/10.6084/m9.figshare.24221227.v1

## Ethical considerations

The principles of the Declaration of Helsinki and Resolution 8430 of Colombia [46] were taken into account for the conduct of the research, according to which the study involves risk-free research.

## Author contributions

Research contribution: Study concept and design, article writing and final approval.

## Conflicts of interest

Sandra Amaya is the Oncology Medical Manager at Astellas Farma Colombia. The remaining authors declare no conflicts of interest.

## Financial disclosure

Sandra Amaya is the oncology medical manager at Astellas Farma Colombia. The remaining authors declare that they have no competing financial interests or known personal relationships that might have influenced the work reported in this paper.

## Funding statement

We declare that this study was funded by Astellas Pharma; however, the literature search, study development, and interpretation of results were conducted independently and objectively by the authors.

## Figures and Tables

**Table 1.** Final cost per patient at nmCRPC stage.

	Presentation	Outline	Tablets/month	Price per mg	Therapy price per month
Enzalutamide	Tablet 40 mg	160 mg/day	120	$2,163.33 COP ($0.55 USD)	$10,383.984 COP ($2,652,24 USD)
Apalutamide	Tablet 60 mg	240 mg/day	120	$1,356.64 COP ($0.35 USD)	$9,767.808 COP ($2,494.86 USD)

COP: Colombian pesos 2022. USD: US dollar^a^

a: The conversion calculations from COP (Colombian pesos) to USD (US dollars) were made using the average representative market rate for the first half of 2022: 1 USD = $3,915.18 COP

Source: Prepared by the authors

**Table 2.** Results of the health value elements for nmCRPC.

Steps						Studies							
						PROSPER, study of enzalutamide plus TPA versus placebo TPA					SPARTAN, study of apalutamide plus TPA versus placebo plus TPA		
						Description			Score		Description		Score
**Step 1: Determine the clinical benefit of the regimen**													
1.A. Is the hazard ratio (HR) of death reported?						HR 0.73 (CI 95%, 0.61–0.89) = 1−0.73 = 0.27*100 = 27			27		HR 0.78 (CI 95%, 0.64–0.96) = 1−0.78 = 0.22*100 = 22		22
1.B. If the HR of death is not reported, is the median OS reported?						Not required					Not required		
1.C. If OS data is not reported, is the HR for disease progression reported?						Not required					Not required		
1.D. If the HR for disease progression is not reported, is PFS reported?						Not required					Not required		
1.E. If the median PFS is not reported, is the RR reported?						Not required					Not required		
1.F. Calculate the clinical benefit score									27				22
**Step 2: Determine the toxicity of the regimen:**													
Does the new regimen represent an improvement in toxicity over the standard of care/comparator?						Enzalutamide = 49.5 Placebo = 38 49.5/38 = 1.3026 1−1.3026 = −0.3026*20 = −6.05 Detail in Appendix 3			-4.64		Apalutamide = 27 Placebo = 22 27/22 = 1.2272 1−1.2272 = −0.2272*20 = −4.54 Detail in Appendix 3		−4.54
**Step 3: Determine the bonus points**													
3.A. Tail of the curve. Identify the time point on the survival curve that is twice the median OS (or PFS) of the comparator regimen. Is there a 50% or greater improvement in the proportion of patients alive with the test regimen at this time (assuming that >20% survive with the standard)?									16				16
3.B. Palliative bonus. Is an improvement in cancer-related symptoms reported?						Not reported			0		Not reported		0
3.C. E-QUOL bonus. Is an improvement in quality of life reported from E-Qol?						Improvement in the subdomain of social and family wellbeing on the FACT-P scale			10		There were no significant differences in quality of life as assessed by EQ-5D-5L and FACT-P		0
3.D. Treatment-free interval bonus. Is the data related to the treatment-free interval reported?						Not reported			0		Not reported		0
3E. Calculate the total bonus points									26				16
**Step 4: Determine the net health benefit of the regimen**						**48.36**					**33.46**		
**Step 5: Determine the cost of the regimen**													
Insert the drug acquisition cost and patient co-payment based on how much the treatment regimen costs per month						$10,383.984 COP ($2,652,24 USD)					$9,767.808 COP ($2,494.86 USD)		
**Step 6: Summary Assessment: Advanced Disease Framework**													
Clinical benefit		Toxicity		Extra points			Net health benefit			Cost of medication per month			
Enza	Apa	Enza	Apa	Enza	Apa		Enza	Apa		Enza		Apa	
27	22	−4.64	−454	26	16		48.36	33.46		$10,383.984 COP ($2,652.24 USD)		$9,767.808 COP ($2,494.86 USD)	

Enza: Enzalutamide. APA: Apalutamide

Source: Prepared by the authors

**Table 3.** Results for the elements of health value for mHSPC.

Steps						Studies							
						ARCHES, study of enzalutamide plus TPA, versus placebo plus TPA					TITAN, study of apalutamide plus TPA, versus placebo plus TPA		
						Description			Score		Description		Score
**Step 1: Determine the clinical benefit of the regimen**													
1.A. Is the hazard ratio (HR) of death reported?						HR 0.66 (CI 95%, 0.53–0.81) = 1−0.66 = 0.34*100 = 34%			34		HR 0.67 (CI 95%, 0.51–0.89) = 1−0.67 = 0.33*100 = 33%		33
1.B. If the HR of death is not reported, is the median OS reported?						Not required					Not required		
1.C. If OS data is not reported, is the HR for disease progression reported?						Not required					Not required		
1.D. If the HR for disease progression is not reported, is PFS reported?						Not required					Not required		
1.E. If the median PFS is not reported, is the RR reported?						Not required					Not required		
1.F. Calculate the clinical benefit score									34				33
**Step 2: Determine the toxicity of the regimen:**													
Does the new regimen represent an improvement in toxicity over the standard of care/comparator?						Enzalutamide = 49.5 Placebo = 45 49.5/45 = 1.1 1−1.1 = −0.11*20 = −2.0 Detail in Appendix 3			-2.0		Apalutamide = 44.5 Placebo = 40 27/22 = 1.11 1−1.1125 = −0.1125*20 = −2.25 Detail in Appendix 3		−2.25
**Step 3: Determine the bonus points**													
3.A. Tail of the curve. Identify the time point on the survival curve that is twice the median OS (or PFS) of the comparator regimen. Is there a 50% or greater improvement in the proportion of patients alive with the test regimen at this time (assuming that >20% survive with the standard)?									0				0
3.B. Palliative bonus. Is an improvement in cancer-related symptoms reported?						Delay of first symptomatic musculoskeletal event HR 0.52 (CI 95%, 0.33–0.80)			10		A statistically significant prolongation in the median time to event for pain deterioration is reported.		10
3.C. E-QUOL bonus. Is an improvement in quality of life reported from E-Qol?									10		No statistically significant differences in global scores or domain scores in the quality of life study are reported		0
3.D. Treatment-free interval bonus. Is the data related to the treatment-free interval reported?						Not reported			0		Not reported		0
3E. Calculate the total bonus points									20				10
**Step 4: Determine the net health benefit of the regimen**						**52**					**40.75**		
**Step 5: Determine the cost of the regimen**													
Insert the drug acquisition cost and patient co-payment based on how much the treatment regimen costs per month						$10,383.984 COP ($2,652,24 USD)					$9,767.808 COP ($2,494.86 USD)		
**Step 6: Summary of the VAF**													
Clinical benefit		Toxicity		Extra points			Net health benefit			Cost of medication per month			
Enza	Apa	Enza	Apa	Enza	Apa		Enza	Apa		Enza		Apa	
34	33	−2.0	−2.25	10	10		52	40.75		$10,383.984 COP ($2,652.24 USD)		$9,767.808 COP ($2,494.86 USD)	

Enza: Enzalutamide. APA: Apalutamide

Source: Prepared by the authors
